# Transgenes of genetically modified animals detected non-invasively via environmental DNA

**DOI:** 10.1371/journal.pone.0249439

**Published:** 2021-08-26

**Authors:** Charles C. Y. Xu, Claire Ramsay, Mitra Cowan, Mehrnoush Dehghani, Paul Lasko, Rowan D. H. Barrett

**Affiliations:** 1 Redpath Museum, McGill University, Montreal, Quebec, Canada; 2 Department of Biology, McGill University, Montreal, Quebec, Canada; 3 McGill Integrated Core for Animal Modeling (MICAM), McGill University, Montreal, Quebec, Canada; University of Hyogo, JAPAN

## Abstract

We demonstrate that simple, non-invasive environmental DNA (eDNA) methods can detect transgenes of genetically modified (GM) animals from terrestrial and aquatic sources in invertebrate and vertebrate systems. We detected transgenic fragments between 82–234 bp through targeted PCR amplification of environmental DNA extracted from food media of GM fruit flies (*Drosophila melanogaster*), feces, urine, and saliva of GM laboratory mice (*Mus musculus*), and aquarium water of GM tetra fish (*Gymnocorymbus ternetzi*). With rapidly growing accessibility of genome-editing technologies such as CRISPR, the prevalence and diversity of GM animals will increase dramatically. GM animals have already been released into the wild with more releases planned in the future. eDNA methods have the potential to address the critical need for sensitive, accurate, and cost-effective detection and monitoring of GM animals and their transgenes in nature.

## Introduction

Environmental DNA (eDNA) is DNA extracted from environmental samples such as soil, sediment, water, air, feces, dust, as well as bulk DNA from artificial and natural collectors like Malaise insect traps, ocean sponges, and spider webs [1–4]. eDNA techniques commonly employ PCR, qPCR, and recently ddPCR to amplify taxonomically informatic DNA markers including 16S and 18S rRNA, cytochrome c oxidase I (COI), and the internal transcribed spacer (ITS) from traces of DNA found in the environment for detection of specific species [5, 6]. Compared to traditional methods, eDNA has proven to be more sensitive and accurate while requiring less time and lower costs [7, 8]. High-throughput next-generation sequencing of DNA markers and shotgun sequencing have also been utilized to generate large genetic data sets that span across taxonomic groups for community-level studies [9–13]. These eDNA methods have revolutionized biodiversity research and are increasingly used by academic biologists, environmental regulatory agencies, and private industry for biomonitoring purposes [14].

In parallel to the development of eDNA methods for biomonitoring, the advent of CRISPR-based genome-editing technologies have revolutionized molecular biology by vastly simplifying the process of creating genetically modified (GM) organisms, which has allowed transgenic research and production to advance dramatically [15]. This sudden democratization of genome-editing is leading to an explosion in the diversity of genetic modifications, the kinds of species targeted, and the contexts in which these methods are applied [16]. For example, do-it-yourself CRISPR kits are currently available for purchase online with little to no restriction [17]. Additionally, CRIPSR-based gene drives have been developed that enable a transgene to quickly spread across a population by favoring the inheritance of the transgene over natural genes [18]. The use of GM animals outside laboratory environments has begun with AquaAdvantage^®^ Atlantic salmon in the aquaculture industry [19]. GM mosquitos have also been released in several locations around the world, and there are plans to release gene-driven GM white-footed mice onto human-populated islands [20–22]. Although the application of GM methods to animal populations in natural settings is expected to increase rapidly in the coming years, there are currently no methods to detect and track GM animals that are efficient, accurate, and sensitive [20, 23].

GM plants have been heavily utilized in agriculture and their transgenes have already been detected from environmental samples [24]. The environment has been found to serve as a reservoir for transgenes from GM plants with short-term persistence (hours to days) in aquatic environments and long-term persistence (days to years) in terrestrial soils [25]. However, to our knowledge, detection of transgenes via eDNA from GM animals in nature has yet to be reported in the literature despite their recent proliferation including insect vectors, livestock, and pets [20]. Because GM animals are indistinguishable from natural individuals based on appearance alone, eDNA methods could be especially useful for early detection and monitoring purposes. Just like wild species, GM animals are expected to shed eDNA through feces, skin cells, decomposition, and other natural processes that can be difficult if not impossible to control. Detectability, persistence, and environmental consequences of animal transgenes left in the environment are still unexplored issues.

**Fig 1:**
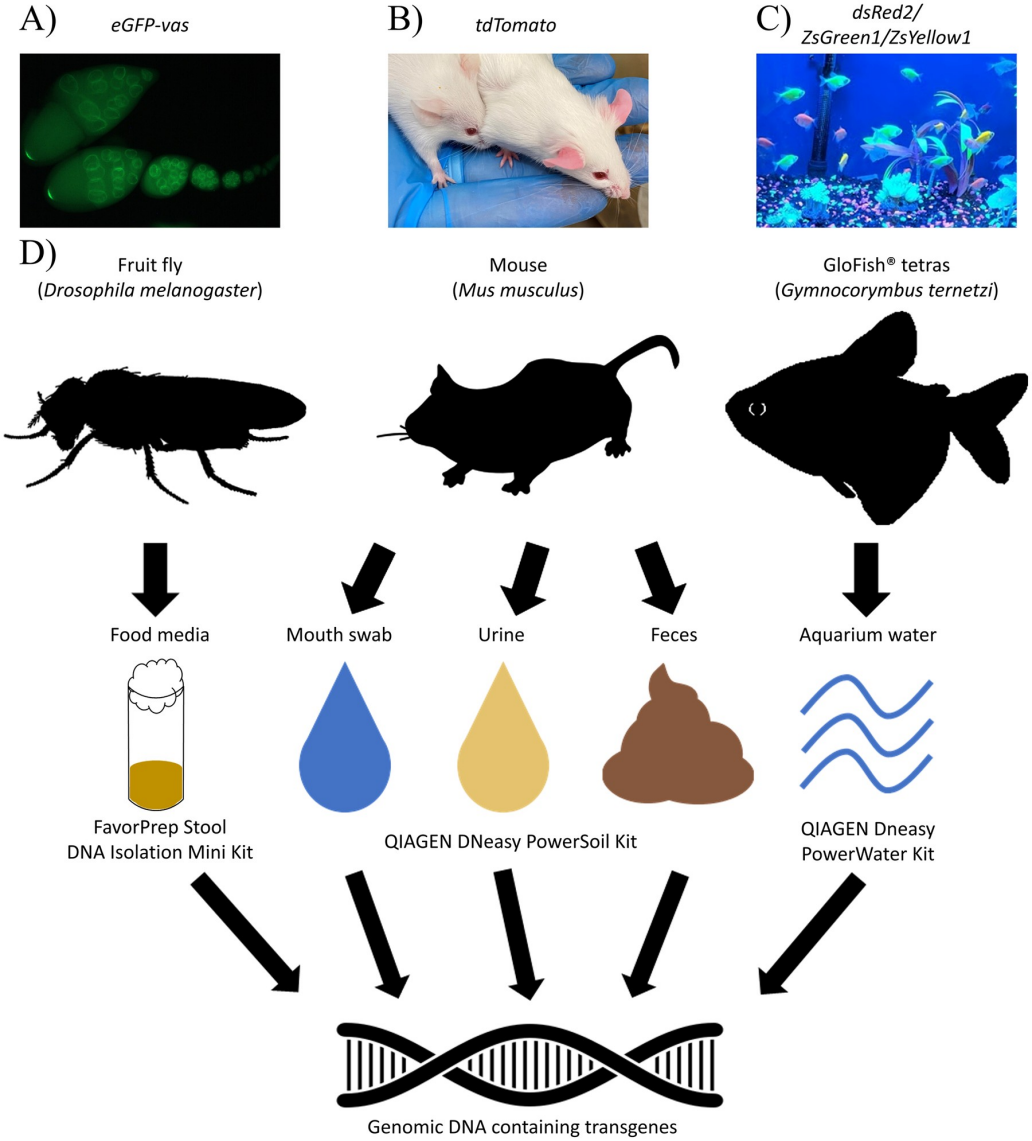
A) Green fluorescent ovary tissue of genetically modified fruit fly (*Drosophila melanogaster*) expressing green fluorescent protein tagged *vasa* gene (*eGFP-vas*). B) Laboratory mice (*Mus musculus*) genetically modified to express *tdTomato* transgene exhibiting reddish skin (right) compared to without *tdTomato* (left). C) Multicolored fluorescent GloFish^®^ tetras (*Gymnocorymbus ternetzi*) expressing combinations of transgenic fluorescence genes in a commercial pet store aquarium. D) Non-invasive environmental DNA samples from a diversity of sources (food media, saliva, urine, feces, and aquarium water) were collected and processed using standard commercial DNA extraction kits.

In this study, we hypothesized that transgenes of GM animals are deposited in their environment and that this extra-organismal DNA could be used to detect the presence of GM animals. We report that fragments of transgenes from GM animals are indeed detectable non-invasively via environmental DNA across three different animal systems: invertebrates (fruit flies; *Drosophila melanogaster*), mammals (laboratory mice; *Mus musculus*), and fish (black tetras; *Gymnocorymbus ternetzi*) (Fig 1).

## Methods

### (a) Sample collection

For the invertebrate system, we extracted eDNA from approximately 3 g of food media from a laboratory fruit fly strain carrying a transgene encoding the green fluorescent protein fused to the *vasa* gene (*eGFP-vas*). The food media contained no observable flies or fly parts. The *eGFP-*tagged full length *vasa* gene was inserted using the *attB/attP* system. We used the FavorPrep Stool DNA Isolation Mini Kit (FAVORGEN Biotech) following the standard protocol except for a 90-minute (instead of 20-minute) incubation at 60°C during the lysis step. We also included an extraction blank using the same extraction method. A positive control from fly tissue was extracted using the following protocol: 1) A single frozen fly was homogenized into buffer containing 10 mM Tris-HCl, 1 mM EDTA, 25 mM NaCl, and 200 μg/mL proteinase K, 2) Fly-buffer mixture was incubated at 37°C for 20 minutes, 3) Supernatant was extracted and incubated at 95°C for 1 minute, 4) DNA was stored at 4°C.

For the mammalian system, we used a laboratory mouse strain carrying the tdTomato transgene (JAX stock number: 007905, Strain Name: B6;129S6-*Gt(ROSA)26Sor*^*tm9(CAG-tdTomato)Hze*^/J) obtained from the McGill Integrated Core for Animal Modeling. We extracted non-invasive extra-organismal DNA from feces inside the housing cage, from ~0.2 mL of urine, and from a cotton oral swab (~ 30 seconds) collected from a single individual. While these samples are technically not true eDNA samples, feces, urine, and saliva are animal eDNA sources in nature and thus provide a useful proof-of-concept since no transgenic mammals have been released to date. DNA extractions were conducted using the DNeasy PowerSoil Kit (QIAGEN) following the standard protocol. We also included an extraction blank and a positive control from an ear punch sample using the same extraction method.

For the fish system, we obtained water from a single 40-gallon aquarium containing approximately 40 GloFish^®^ Cosmic Blue^®^, Electric Green^®^, Galactic Purple^®^, Moonrise Pink^®^, Starfire Red^®^, and Sunburst Orange^®^ tetras (GloFish LLC, hereafter called GloFish tetras) from a local pet store (Montreal, Quebec, Canada). We filtered approximately one liter of aquarium water through 0.22 μM and 0.7 μM polyethersulfone filter papers (Millipore) separately using a handpump (Mityvac). Both filter pore sizes were used to maximize detection probability since the particle size of transgenic eDNA is unknown. We extracted eDNA from filter papers using the DNeasy PowerWater Kit (QIAGEN) following the standard protocol. We also included an extraction blank using the same extraction method.

All sample collection was non-invasive and did not involve any entire living materials. This “A” level of invasiveness did not require animal use approval at McGill University.

### (b) Primer design

We designed three different sets of primers to amplify 82–187 bp of the *eGFP* gene for detection of GM fruit flies (Table 1). A single pair of primers were used to amplify a 196 bp fragment of the *tdTomato* gene from GM laboratory mice (Table 1). For detection of GM GloFish tetras, we designed three sets of primers targeting: 1) 213 bp of *dsRed2*, 2) 210 bp of *ZsGreen1*, and 3) 234 bp of *ZsYellow1* fluorescent genes [26] (Table 1). All primers were designed based on publicly available sequences obtained from the National Center for Biotechnology Information (NCBI) GenBank database using the Primer3 software [27, 28].

**Table 1:** Primers for PCR amplification of transgenic elements.

Name	Sequence (5’-3’)	Forward/Reverse	Length (bp)	Tm (C°)	GC (%)	Target	Amplicon size (bp)	Reference
egfp_F1	GAGCAAAGACCCCAACGAGA	Forward	20	59.97	55	eGFP	82	This study
egfp_R1	GTCCATGCCGAGAGTGATCC	Reverse	20	60	60	eGFP		This study
egfp_F2	ACGTAAACGGCCACAAGTTC	Forward	20	60	50	eGFP	187	This study
egfp_R2	AAGTCGTGCTGCTTCATGTG	Reverse	20	60.1	50	eGFP		This study
egfp_F3	TATATCATGGCCGACAAGCA	Forward	20	60.1	45	eGFP	163	This study
egfp_R3	ACTGGGTGCTCAGGTAGTGG	Reverse	20	60.2	60	eGFP		This study
oIMR9103	GGCATTAAAGCAGCGTATCC	Forward	20	60.5	50	tdTomato	196	[54]
oIMR9105	CTGTTCCTGTACGGCATGG	Reverse	19	60.5	57.9	tdTomato		[54]
dsRed2_F1	GAACGTCATCACCGAGTTCA	Forward	20	59.7	50	dsRed2	213	This study
dsRed2_R1	GGGTGCTTCACGTACACCTT	Reverse	20	60	55	dsRed2		This study
ZsGreen1_F1	CCCCGTGATGAAGAAGATGA	Forward	20	61	50	ZsGreen1	210	This study
ZsGreen1_R1	GTCAGCTTGTGCTGGATGAA	Reverse	20	60	50	ZsGreen1		This study
ZsYellow1_F1	GACCGGATCTTCACCGAGTA	Forward	20	60.1	55	ZsYellow1	234	This study
ZsYellow1_R1	CTCCCAGTTGGTGGTCATCT	Reverse	20	60	55	ZsYellow1		This study

### (c) PCR amplification and analysis of products

DNA concentrations of samples were quantified using the Quant-iT^™^ High-Sensitivity dsDNA Assay (Invitrogen). DNA samples were amplified in polymerase chain reactions (PCR) of 10 μL containing 6.36 μL of ultrapure water (Milli-Q), 1 μL of 10X PCR Buffer (Invitrogen), 0.3 μL of 50 mM MgCl_2_ (Invitrogen), 0.3 μL of 10 mM dNTP Mix (Invitrogen), 0.5 μL 10 mM forward primer, 0.5 μL 10 mM reverse primer, 0.04 μL of Platinum© Taq DNA Polymerase (Invitrogen), and 1 μL (<0.2–102.16 ng/μL) of genomic DNA. Negative control reactions with ultrapure water instead of DNA were included in every PCR to test for contamination. Gel electrophoresis was conducted using 5 μL of PCR product mixed with 1 μL TriTrack DNA Loading Dye (6X) (Thermo Scientific) and amplicon length was estimated using GeneRuler 100 bp DNA ladder (Thermo Scientific). Bi-directional Sanger sequencing was conducted on an ABI 3730xl 96-capillary sequencer by the Centre d’expertise et de services Génome Québec. DNA sequences were aligned using BioEdit v.7.2.5 and ClustalW [29, 30].

## Results

**Fig 2:**
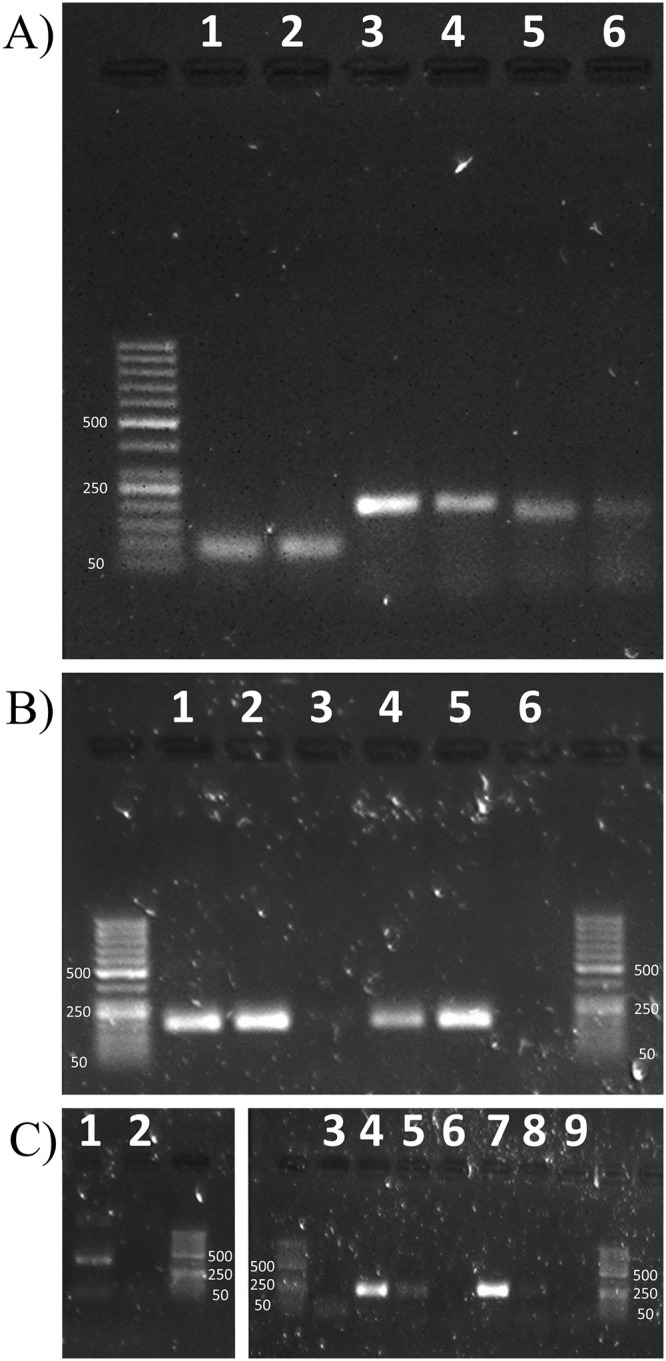
Agarose gel electrophoresis showing A) amplification of *egfp* from genetically modified fruit fly (*Drosophila melanogaster*)– 1: fly tissue with egfp_F1/R1, 2: fly food media with egfp_F1/R1, 3: fly tissue with egfp_F2/R2, 4: fly food media with egfp_F2/R2, 5: fly tissue with egfp_F3/R3, 6: fly food media with egfp_F3/R3, B) amplification of *tdTomato* from feces, urine, and saliva of genetically modified mouse (*Mus musculus*)– 1,2: mouse feces from different cages, 3: mouse urine (weak amplification), 4: mouse mouth swab, 5: mouse ear punch, 6: DNA extraction negative control (no amplification), and C) amplification of *dsRed2*, *ZsGreen1*, and *ZsYellow1* from filtered aquarium water of GloFish^®^ tetras (*Gymnocorymbus ternetzi*)– 1: 0.22 μM filtered water with dsRed2 F1/R1, 2: 0.7 μM filtered water with dsRed2 F1/R1 (no amplification), 3: DNA extraction negative control (no amplification), 4: 0.22 μM filtered water with ZsGreen1 F1/R1, 5: 0.7 μM filtered water with ZsGreen1 F1/R1 (weak amplification, 6: DNA extraction negative control (no amplification), 7: 0.22 μM filtered water with ZsYellow1 F1/R1, 8: 0.7 μM filtered water with ZsYellow1 F1/R1 (weak amplification), 9: DNA extraction negative controls (no amplification). Gel images were captured using Quantum Vilber Lourmat (MBI) and were cropped to only relevant lanes.

Genomic DNA concentrations of eDNA extractions ranged from <0.2 ng/μL (threshold of quantification assay) to 102.16 ng/μL. All target transgenes were successfully detected based on estimated amplicon sizes except for *dsRed2* from the 0.7 μM filter while extraction blanks and PCR negative controls yielded no amplification (Fig 2). DNA sequences were obtained via Sanger sequencing. Forward and reverse reads of each sample were aligned, and primer sequences were then removed. Transgene identities of aligned amplicons were confirmed by NCBI BLAST using default settings and alignment with reference genes downloaded from the GenBank Nucleotide database [31]. All raw DNA sequences and reference alignments are accessible on DRYAD at https://doi.org/10.5061/dryad.866t1g1pp.

## Discussion

Our results demonstrate that transgenes from a diversity of GM animals can be detected from non-invasive environmental DNA samples thus providing proof-of-concept that eDNA has the potential to be a powerful tool in biomonitoring of GM animals. The single failed amplification of *dsRed2* from the 0.7 μM filter is likely due to low total DNA concentration (<0.2 ng/μL), which is consistent with weak amplification of *ZsGreen1* and *ZsYellow1* from the same sample. Despite DNA concentrations of less than the threshold of the quantification assay, the 0.7 μM filter along with the mouse urine and mouth swab samples still successfully amplified and produced clear chromatograms from Sanger sequencing suggesting that eDNA methods are highly sensitive in detecting transgenes. Our results also suggest that transgenes are more likely to be detected using 0.22 μM rather than 0.7 μM filters in aquatic environments. While both mouse urine and mouth swab samples yielded less than quantifiable amounts of total DNA, only the urine sample showed weak amplification, which indicates that concentration of transgenic DNA may not always correspond with total DNA concentration. This relationship is predicted to change depending on the type of eDNA sample collected and the amount of nontarget DNA present [25].

The samples used in this study were collected under laboratory conditions and commercial settings, which likely biased detection success. Application of eDNA methods for detection of transgenes from GM animals in nature is expected to be more complicated due to environmental exposure and fluctuating conditions [32, 33]. Typical eDNA assays target short gene fragments because eDNA is readily susceptible to degradation, influenced by factors such as temperature, turbidity, acidity, salinity, and bacterial abundance [34]. Determining the particle size, degradation, persistence, and ecological fate of animal transgenes in the environment will be important in developing eDNA methods for tracking GM animals [25, 35, 36]. Nonetheless, this proof-of-concept demonstration is the first step towards future validation studies conducted in field settings using more sensitive methods such as qPCR and ddPCR. Metabarcoding and metagenomic methods also hold promise for simultaneous detection of multiple transgenes across multiple GM species [37].

One important factor affecting the sensitivity of eDNA methods is the copy number of the target DNA sequence. Most eDNA studies use mitochondrial DNA like 16S rRNA or the COI gene to maximize detection probability because of their high copy numbers per cell [38]. Additionally, eDNA studies using multicopy nuclear genes like 18S rRNA and ITS have also been successful [39]. While some transgenes are present in tandem multiple copy arrays across the nuclear genome, many are single genes that have either been edited or inserted [40]. Single transgenes may thus be relatively harder to detect than conventional eDNA markers due to copy number differences. Additionally, if the eDNA detection method targets a specific transgenic allele, genotype may also influence sensitivity (homozygous allele copy number is twice that of heterozygous and hemizygous alleles in diploid species) [41]. Transgenes are also often inserted inside transposons, which can lead to multiple independent insertion events and positively bias eDNA detection. Another unexplored research frontier is the consequence of newly available epigenome-editing tools on the efficiency of eDNA amplification and sequencing of epigenetically modified genes due to potential structural changes [42].

Concerns have been raised about the potential for transmission of transgenes from GM organisms and the subsequent ecological effects. Methods of transmission into unintended populations and species include cross-pollination, hybridization, and horizontal gene transfer (HGT) [43]. For example, despite the presence of a dominant lethal transgene, reportedly sterile GM mosquitoes in Brazil have been able to create viable hybrids with wild individuals [44]. Additionally, there are demonstrated ecological impacts of viable hybrids created from GM Atlantic salmon and wild brown trout, which are able to grow faster and competitively suppress both GM and wild salmon [45]. HGT through a natural ability to uptake naked plasmids and fragments of chromosomal DNA directly from the environment has been observed in many bacterial species across a variety of habitats [46]. While there has been no documented case of HGT from GM animals in nature, there is evidence for HGT of transgenes from GM plants to bacteria and fungi despite transmission and establishment barriers (although these events are rare and mostly limited to transgenes of bacterial origin that are often already abundant in the environment) [47]. Despite these valid concerns, GM organisms have many significant benefits for the environment, human health, agriculture, and industry that have improved global human well-being and have led to valuable scientific discoveries [20].

The advantages of using eDNA to detect GM organisms could synergize well with artificial DNA barcodes. Used as identification tags for transgenes, artificial DNA barcodes can be synthesized to contain a unique DNA sequence not found in nature [48, 49]. These silent barcodes are neither transcribed nor translated and their sole purpose is to track neighboring transgenes. Artificial DNA barcodes can be linked to metadata associated with the barcoded GM individual (e.g., identities and number of transgenes present, geographic location and date of creation, intended usage, etc.), and multiple barcodes within a single individual can also be used to independently track multiple transgenes using a metagenomics approach. The design of artificial DNA barcodes would incorporate primer binding sites to facilitate efficient eDNA detection, enabling sensitive, non-invasive, and ubiquitous biomonitoring of GM organisms. By providing a method for quick and easy differentiation of GM organisms, artificial DNA barcodes may help to alleviate public and governmental concerns and inform policies regarding their potential release. In addition, artificial DNA barcodes may be incorporated into gene-drives to track their spread across populations, which has been a major concern for application of gene-drives in nature [50]. Although the idea of artificial DNA barcodes is not new, and they have been used to ‘watermark’ artificially synthesized genomes, we are unaware of wide adoption by regulatory agencies or industry [51–53]. Further development of biotechnologies like artificial DNA barcodes and their use with emerging biomonitoring methods like eDNA could become an important tool for transgenic producers and regulators to mitigate potential environmental and human health risks of creating and releasing GM animals.

## Conclusion

Potential escape of GM animals from their intended locations and potential introgression of transgenes into unintended populations and species could have significant ecological, evolutionary, and bioethical implications. eDNA methods will improve our ability to locate and manage released GM animals and their transgenes across diverse species and environments in these scenarios.

## Supporting information

S1 Raw images (ZIP)
