# Supplementary figures and images for: Transgenes of genetically modified animals detected non-invasively via environmental DNA
Source: PLoS One. 2021 Aug 26;16(8):e0249439. doi: 10.1371/journal.pone.0249439 (PMC8389434; doi:10.1371/journal.pone.0249439)

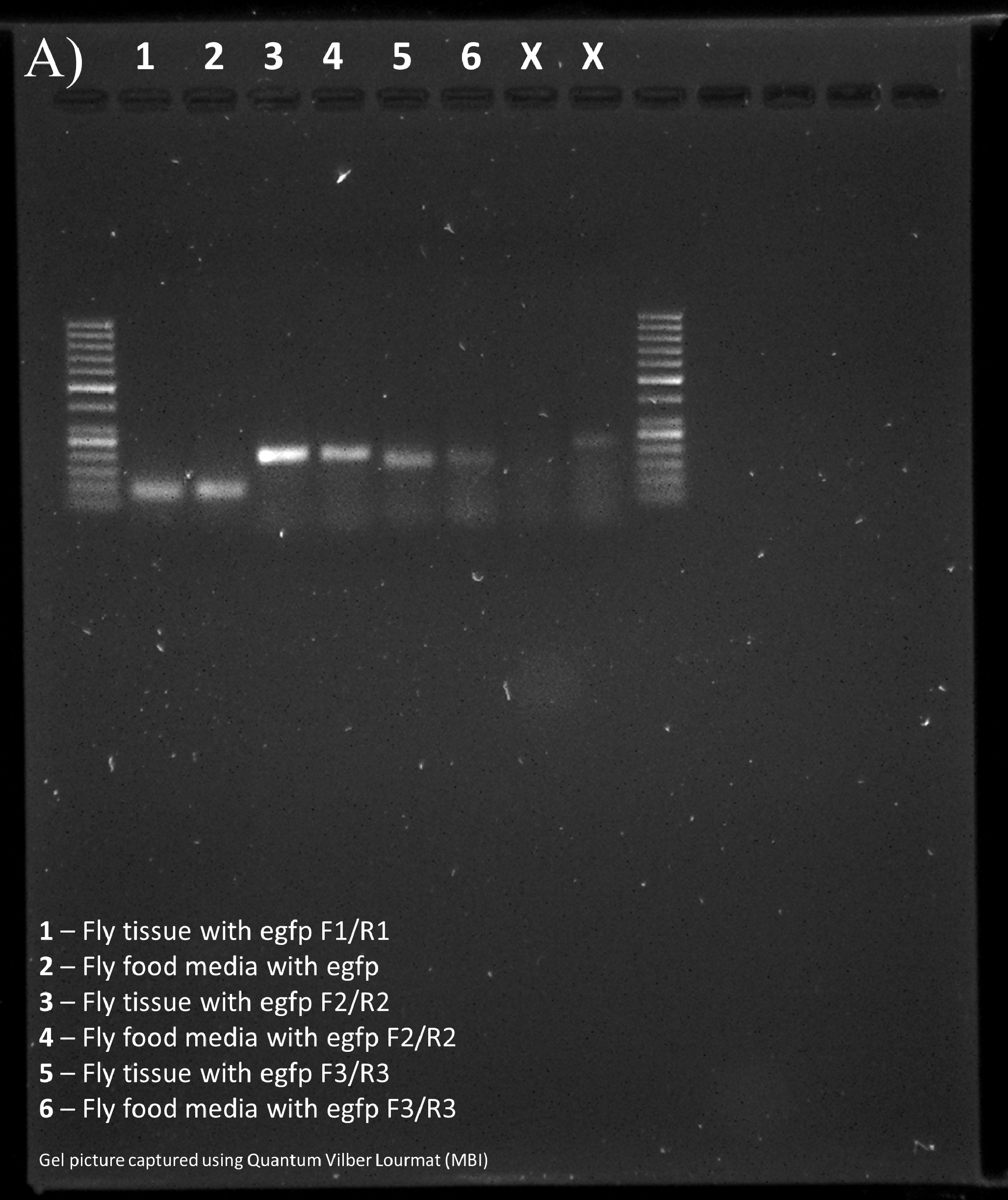

Supplement: S1 Raw images — (ZIP) [file pone.0249439.s001.zip › S1_raw_image_A.tif]

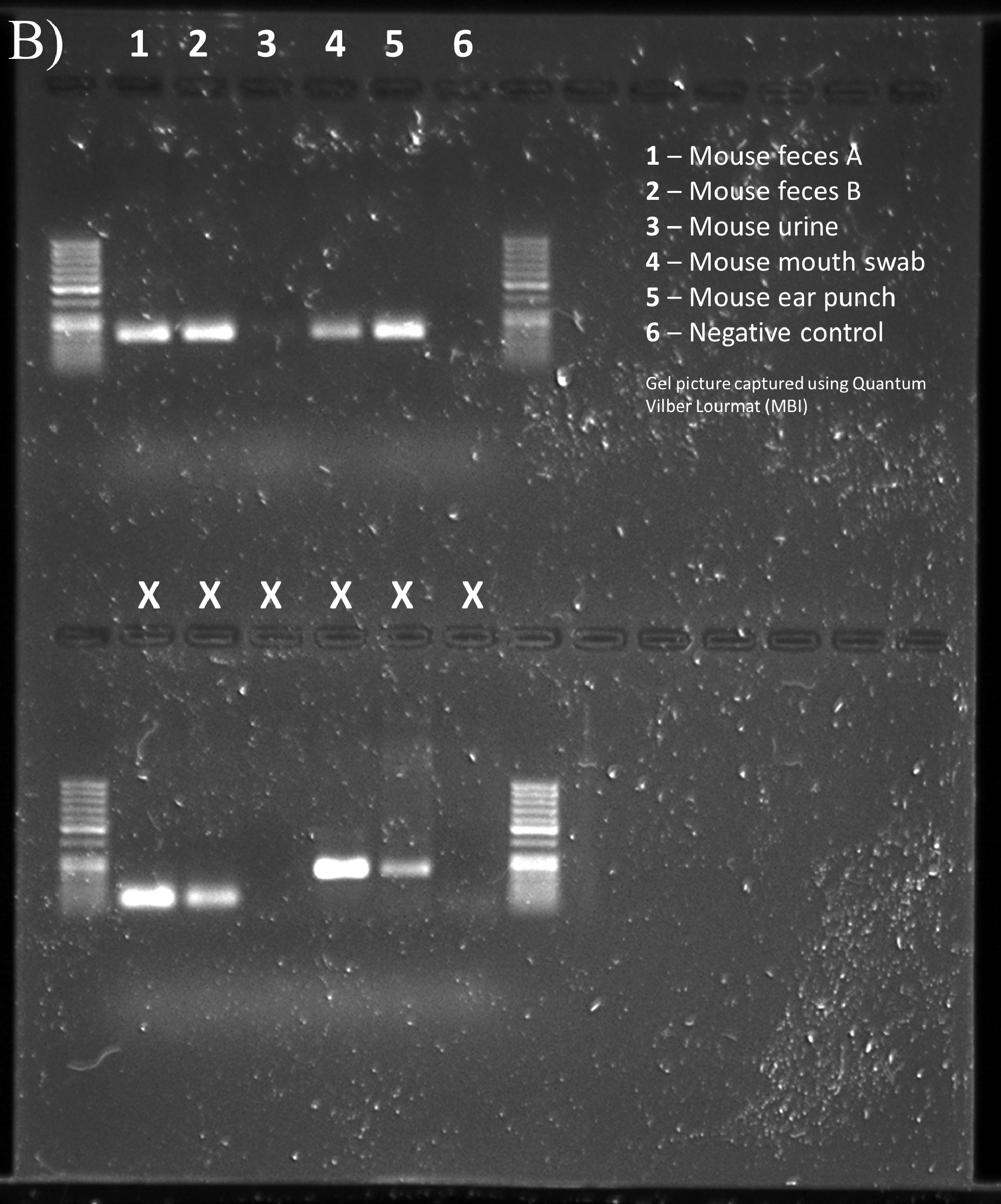

Supplement: S1 Raw images — (ZIP) [file pone.0249439.s001.zip › S1_raw_image_B.tif]

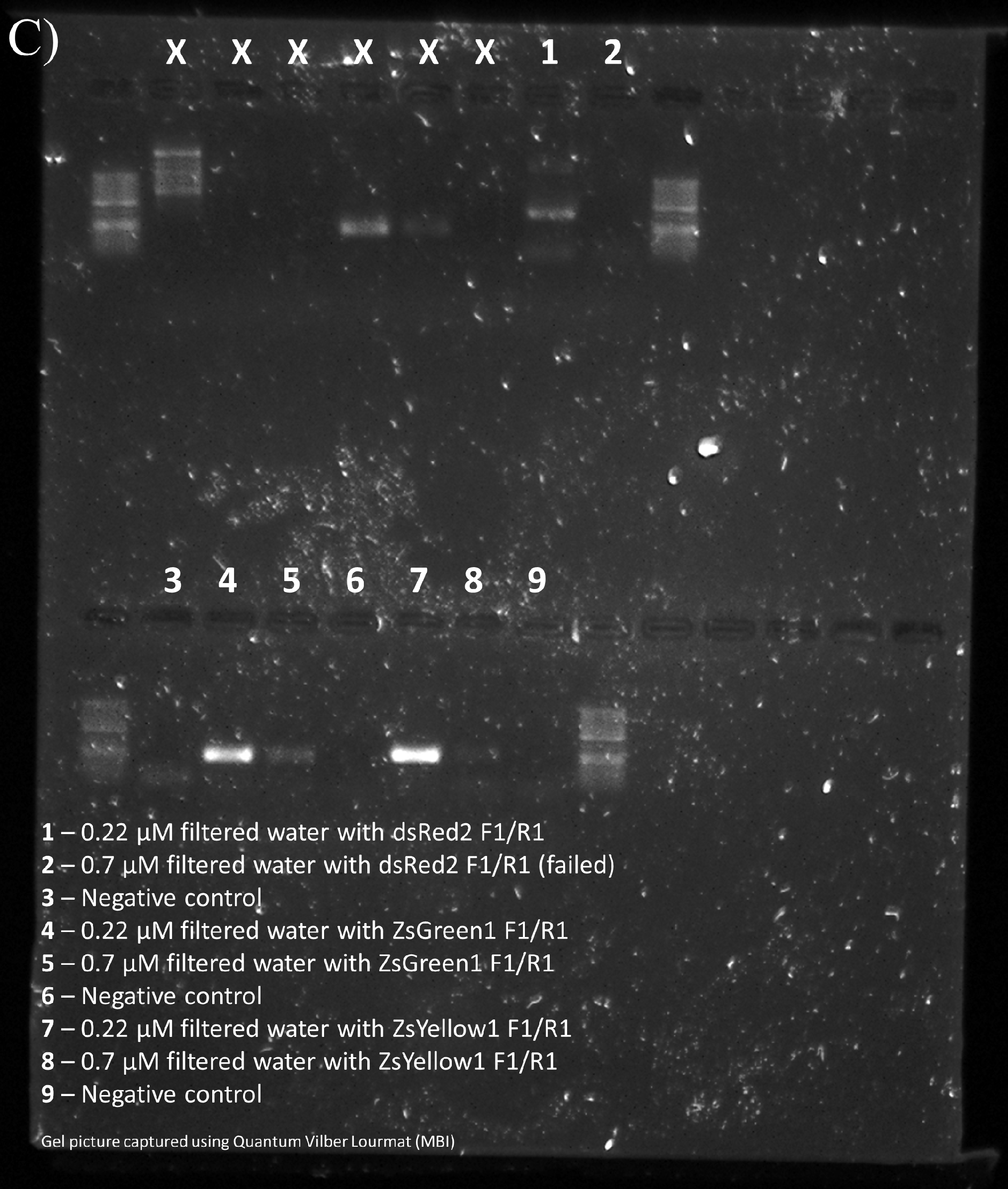

Supplement: S1 Raw images — (ZIP) [file pone.0249439.s001.zip › S1_raw_image_C.tif]
